# Antimicrobial Stewardship for a Geriatric Behavioral Health Population

**DOI:** 10.3390/antibiotics5010008

**Published:** 2016-01-19

**Authors:** Kristen Ellis, Georgina Rubal-Peace, Victoria Chang, Eva Liang, Nicolas Wong, Stephanie Campbell

**Affiliations:** 1 Banner Medical University Center South, 2800 E Ajo Way, Tucson, AZ 85713, USA; georgina.rubal-peace@bannerhealth.com; 2 University of Arizona College of Pharmacy, 1295 N Martin PO Box 210202, Tucson, AZ 85721-0202, USA; chang@pharmacy.arizona.edu (V.C.); liang@pharmacy.arizona.edu (E.L.); nwong@pharmacy.arizona.edu (N.W.); scampbell@pharmacy.arizona.edu (S.C.)

**Keywords:** antimicrobial stewardship, pharmacist, geriatric, mental health

## Abstract

Antimicrobial resistance is a growing public health concern. Antimicrobial stewardship and multi-disciplinary intervention can prevent inappropriate antimicrobial use and improve patient care. Special populations, especially older adults and patients with mental health disorders, can be particularly in need of such intervention. The purpose of this project was to assess the impact of pharmacist intervention on appropriateness of antimicrobial prescribing on a geriatric psychiatric unit (GPU). Patients ≥18 years old prescribed oral antibiotics during GPU admission were included. Antimicrobial appropriateness was assessed pre- and post-pharmacist intervention. During the six-month pre- and post-intervention phase, 63 and 70 patients prescribed antibiotics were identified, respectively. Subjects in the post-intervention group had significantly less inappropriate doses for indication compared to the pre-intervention group (10.6% *vs.* 23.9%, *p* = 0.02), and significantly less antibiotics prescribed for an inappropriate duration (15.8% *vs.* 32.4%, *p* < 0.01). There were no significant differences for use of appropriate drug for indication or appropriate dose for renal function between groups. Significantly more patients in the post intervention group had medications prescribed with appropriate dose, duration, and indication (51% *vs.* 66%, *p* = 0.04). Pharmacist intervention was associated with decreased rates of inappropriate antimicrobial prescribing on a geriatric psychiatric unit.

## 1. Introduction

Adults over 65 years in age are much more likely to be taking multiple medications than younger adults, and are approximately seven times more likely to be hospitalized for an adverse drug reaction [1]. High rates of medication errors at hospital admission have also been reported for patients with mental health disorders [2]. Antimicrobials are commonly prescribed inappropriately, and rising rates of antimicrobial resistance is a serious public health threat [3]. In addition, *Clostridium difficile* infection (CDI) was estimated to cause $3.2 billion in excess healthcare costs in 2000–2002 [4,5]. In an era in which antimicrobial drug resistance, adverse drug reactions, and CDI is increasingly prevalent, it is important for a multidisciplinary team to work together to tailor the most appropriate antibiotic regimen, especially for vulnerable populations.

Pharmacists can be a valuable addition to the healthcare team and play an important role in ensuring antibiotic appropriateness. Murray *et al.* conducted a prospective before-and-after study that focused on inappropriate duration of antibiotic therapies used for respiratory tract infections [6]. They found that implementation of required stop dates and utilization of pharmacists’ interventions was associated with decreased antibiotic duration by 18.1% and decreased adverse effects by 19%. Additionally, a systematic review and meta-analysis evaluated the impact of pharmacists on outcomes for geriatric patients and demonstrated that pharmacists improved drug therapy, safety, adherence, and reduced hospitalization [7]. This analysis included 20 studies ranging from 36 to 4218 subjects with an average age of 65 years, and was conducted in ambulatory and inpatient care settings.

This project was performed at a 245 bed academic medical center that currently has no formal antimicrobial stewardship program in place. In addition to critical care and internal medicine services, this medical center is a major provider of behavioral health services to the area and has an 18 bed geriatric psychiatric unit (GPU). Patients are admitted to this floor only once they have been medically cleared, and often have an extended length of stay. It is common that patients are transferred to this floor while completing a course of antibiotics, have antibiotics started in the emergency room prior to admission, or initiate antibiotics during their extended admission. In addition, due to the association between infection and altered mental status in older adults, antibiotics are often empirically initiated upon admission to the GPU. During a six-month period 280 patients were admitted to the GPU and 22.5% of these patients were prescribed an antibiotic. It was observed that these antibiotics were often continued without a designated end date or an appropriate indication. Appropriate antibiotic usage is pertinent to preventing antimicrobial resistance. It was found that fluoroquinolone susceptibility at our facility overall had decreased from 2013 to 2014. Specifically, we saw a decrease in ciprofloxacin susceptibility for *Escherichia coli*, *Pseudomonas aeruginosa*, and *Proteus mirabilis*. In addition, susceptibility of oxacillin sensitive *Staphylococcus aureus* to levofloxacin decreased. While previous studies have demonstrated that pharmacists can have an impact on appropriate antibiotic use, there is a lack of data in regards to antimicrobial stewardship in the geriatric behavioral health population. We set out to attempt a multidisciplinary approach, via additional pharmacist intervention, in order to improve antimicrobial prescribing on the GPU. The objective of this quality improvement (QI) project was to investigate the impact of pharmacy implementation of an antimicrobial stewardship intervention on antibiotic appropriateness on the GPU.

## 2. Results and Discussion

### 2.1. Patient Demographics

During the six-month pre- and post-intervention phases, there were 63 and 70 patients who met inclusion criteria, respectively. Baseline characteristics were compared between groups and recorded in Table 1. The average ages for pre-intervention and post-intervention groups were 66.16 years and 65.68 years, respectively, which was not statistically significantly different. There was a significant difference between groups in regard to gender, with the pre-intervention group consisting of a significantly greater proportion of females. There were also differences at baseline between groups in the number of patients with renal impairment and number of patients prescribed multiple antibiotics. The pre-intervention group had significantly more patients with renal impairment, and the post-intervention group had significantly more patients prescribed multiple antibiotics. A binary logistic regression of the data revealed that the differences at baseline were marginal and had limited impact on antibiotic appropriateness.

Urinary tract infection was the most common treatment indication in both groups, accounting for greater than 50% of infections identified. Cellulitis was the second most common indication, followed by a compilation of “other” diagnoses. Indications identified in the “other” category were: dental abscesses, chronic obstructive pulmonary disease (COPD) exacerbations, pneumonia, and acute bronchitis. Ciprofloxacin and cephalexin were the most commonly prescribed antimicrobials in both groups, and also the most commonly inappropriately prescribed antimicrobials in both groups.

**Table 1:** Baseline Demographics.

Characteristic	Pre (*n* = 63)	Post (*n* = 70)
Mean age (standard deviation), years	66.16 (12)	65.68 (13.7)
Females ^+^ *n* (%)	51 (81)	45 (64.3)
Patients with multiple antibiotics ^+^ *n* (%)	8 (12.7)	19 (27.1)
Patients with renal impairment *^,+^ *n* (%)	44 (69.8)	21 (30)
Type of Infection, *n* (%)		
UTI	49 (77.8)	51 (65.4)
Cellulitis	5 (7.9)	5 (6.4)
Other	9 (14.3)	22 (28.2)

* Renal impairment defined as CrCl < 60 mL/min; ^+^ significant difference between the groups (*p* < 0.05).

### 2.2. Antimicrobial Appropriateness

Antimicrobial appropriateness, including correct drug for indication, correct dose for indication, correct dose for renal function if impairment is present, and correct antimicrobial duration were assessed (Table 2). There were 71 antimicrobials prescribed in the pre-intervention group and 95 antimicrobials in the post-intervention group. There were no differences in whether antimicrobial choice was appropriate for indication between groups (*p* = 0.48). Specifically, 21.1% (pre-intervention) and 16.8% (post-intervention) of the antimicrobials prescribed were an inappropriate drug choice for indication. However, 23.9% of the dosing in the pre-intervention group was inappropriate for indication, and after pharmacist intervention, there was a statistically significant decrease in inappropriate antibiotic dose to 10.5% (*p* = 0.02). Rates of inappropriate dosing for renal function were improved in the post intervention group compared to the pre-intervention group, but this result was not statistically significant (11% compared to 36%, *p* = 0.11). Pre-intervention and post-intervention comparison of antimicrobial duration appropriateness found a significant improvement from 32.4% to 15.8% post intervention (*p* = 0.01). Comparison of a composite of appropriate medication for indication, appropriate dose for indication, appropriate dose for renal function, and appropriate duration for indication demonstrated a greater number of patients in the post-intervention group met criteria for appropriateness in all four categories compared to patients in the pre-intervention group (66% compared to 51%, *p* = 0.04). There was one incident of CDI in the post-intervention phase.

**Table 2:** Antimicrobial Use Results.

Outcome	Pre (*n* = 71)	Post (*n* = 95)	*p*-Value
Appropriate medication for indication	56 (79%)	79 (83%)	0.48
Appropriate dose for indication	54 (76%)	85 (89%)	0.02
Dose renally adjusted *	18 (64%)	17 (89%)	0.11
Appropriate duration for indication	48 (68%)	80 (84%)	0.01
Pharmacist intervention	5 (7%)	34 (36%)	<0.01
Appropriate medication, dose, duration, renal adjustment	36 (51%)	63 (66%)	0.04

* Excluded: antibiotics prescribed to patients with normal renal function, patients in which renal function was unknown, andantibiotics that did not require renal dose adjustment.

Overall, the pharmacist intervened and made recommendations for therapy adjustments in 7% of antimicrobials prescribed in the pre-intervention group, and 35.8% of antimicrobials prescribed in the post intervention group (*p* < 0.01). Prior to the intervention phase, a clinical pharmacist was not typically reviewing antibiotic prescribing on the GPU. There were five interventions performed in the pre-intervention phase by pharmacy. These interventions occurred during order verification or if the physician directly called pharmacy to ask a question. As a result, most orders were not comprehensively reviewed. Although all orders were verified by a pharmacist, this was typically a limited review of dose, drug interactions, and allergies. In addition, there was often little information available (cultures, labs, history) during initial verification.

Duration of therapy normalized per 1000 patient days (DOT/1000 patient days) was also calculated to assess differences between antimicrobial utilization between groups. The DOT/1000 patient days for the pre- and post-intervention groups was 174.2 and 174.7, respectively (*p* = 0.99).

## 3. Materials and Methods

### 3.1. Project Ethical Considerations

Prior to initiation, an Institutional Review Board (IRB) form 309 (Human Research Determination) was completed for this quality improvement (QI) project and the project was deemed non-human subjects research, designed to enhance patient care through implementation of an evidence-based expanded service in a particular setting, and exempt from University of Arizona IRB approval.

### 3.2. Intervention

A non-randomized pre-post-intervention study design was used for this retrospective analysis. During the 6 month intervention period (1 August 2014 through 31 January 2015), a clinical pharmacist used the electronic medical record (EMR) to run a weekly report of all patients on the GPU prescribed oral antimicrobials. The report was designed to identify all formulary oral antimicrobials, including moxifloxacin, levofloxacin, ciprofloxacin, cephalexin, sulfamethoxazole/trimethoprim, nitrofurantoin, amoxicillin/clavulanic acid, amoxicillin, azithromycin, metronidazole, clarithromycin, vancomycin, clindamycin, doxycycline, cefuroxime, and dicloxacillin. Once patients were identified, the patient chart was reviewed, and therapy appropriateness was evaluated.

Therapy evaluation included assessment of antimicrobial choice for indication or cultures, assessment of antimicrobial dose for indication, assessment of antimicrobial renal dose adjustment in patients with renal impairment, and assessment of antimicrobial duration. Assessments were based upon Infectious Diseases Society of America (IDSA) guidelines and the Global Initiative for Chronic Obstructive Lung Disease (GOLD) guidelines for appropriatene Type of Infection, *n* (%)ss of drug selection and duration [8,9,10,11]. Physician documentation in the medical record and/or culture results were used to determine medication indication and classification of infection severity. Based on this classification, antibiotic choice for indication was deemed appropriate if the choice was listed by IDSA or GOLD guidelines as an appropriate option, taking in to account patient allergy information. Antibiotic dose appropriateness was assessed using Lexicomp Drug Information Reference, and physician classification of severity was used to determine appropriate dosage ranges for antibiotics with multiple doses. The dose was deemed appropriate if it fell within the range recommended per the Lexicomp drug information reference and was appropriately renally dosed according to this reference. If the therapy was considered inappropriate, the pharmacist made therapy adjustment recommendations to the prescribing physician, and documented the intervention in the patient’s chart. When recommending antibiotic duration, stop dates were placed on antimicrobial orders.

### 3.3. Data Collection

Patients were included in the project if they were ≥18 years old, were admitted to the GPU between 1 February 2014 through 31 July 2014 or 1 August 2014 through 31 January 2015, and were prescribed oral antibiotics during their admission to this unit. Reports for all oral antibiotics prescribed during the project period were generated. The reports were separated into pre-intervention (February–July 2014) and post-intervention groups (August 2014–January 2015), respectively. Data was collected retrospectively from the EMR using a standardized data collection form. We recorded the following baseline demographics for each patient: age, gender, number of antibiotics prescribed, type of infection and renal function. We defined renal impairment as a creatinine clearance of less than 60 mL/min using the Cockcroft-Gault equation. Type of infection was further categorized as a urinary tract infection (UTI), cellulitis, or other. The “other” category consisted of indications that did not have a high frequency of occurrence throughout the data collection process. For analysis of the dependent variable, antibiotic appropriateness was separated into five different categories and collected as nominal data. These categories included correct antibiotic for indication, dose for indication, renal adjustments, duration, and pharmacy intervention. Microbiologic culture results and urinalysis results were also collected when available. Physician documentation in progress notes for indication were also recorded. Finally, duration of therapy data was obtained from Pyxis archives and census data for the GPU was used to acquire information about total patient days during the study period.

### 3.4. Data Analysis

Descriptive statistics were used to describe demographic data. Fisher’s exact test was used to evaluate baseline differences between the pre and post-intervention groups for nominal data. Yate’s correction factor was used if a frequency of any cell was less than five. An independent t-test was used to compare patient age between the pre and post-intervention groups. The alpha level was set to 0.05. We used the Statistical Package for the Social Sciences (SPSS) software to run multiple logistic regressions to correct for baseline differences. The total duration of therapy (DOT) was calculated and normalized for 1000 patient days.

## 4. Conclusions

### 4.1. Conclusion

This project constitutes an example of a multi-disciplinary intervention strategy for improving antimicrobial prescribing in the absence of a formal antimicrobial stewardship program. Specifically, this intervention had a positive impact on evidence-based selection of antimicrobial duration and dosing for a geriatric psychiatric population. Our results highlight the need for specific attention to renal dose adjustment and appropriate management of urinary tract infection when reviewing antimicrobial prescribing in a geriatric population. For facilities where formal antimicrobial stewardship has not been established, this project also exemplifies how electronic medical record (EMR) generated reports may assist in antimicrobial management.

### 4.2. Limitations

There were several limitations to this project. The data was retrospectively extracted from the medical record in a non-blinded manner, and accurate documentation was assumed. Due to the retrospective nature of the project, some data was not available for all patients. If information was missing that was necessary for assessing appropriateness, the item was coded as inappropriate dose, duration, or indication. For renal dose adjustment, appropriateness of dose was not assessed if serum creatinine values were not available.

UTI was the most commonly treated infection in this project. Based upon evidence that screening for and treating asymptomatic bacteriuria in elderly institutionalized subjects is of little benefit, intervention did include questions about presence of urinary symptoms [12]. However, it was common for patients admitted to the GPU to present with altered mental status (AMS). Urinary tract infections, in particular, are common causes of geriatric mental status changes, and AMS may be the only identifiable symptom in some cases. In the case that UTI was considered a possible cause of AMS on admission, patients were often treated empirically and a urinalysis was collected. When asked about asymptomatic bacteriuria, it was difficult for physicians to differentiate between AMS related to UTI from AMS related to other causes, and therefore antibiotics were often continued. Therefore, it is possible that cases of asymptomatic bacteriuria were treated with antibiotics. This issue, along with the inability of some older adults to communicate whether symptoms are present, is a potential cause of antibiotic overprescribing in this population.

The study focused on quality of antibiotic use within hospital stay and our data and results reflect this. We were unable to assess for many clinical outcomes, such hospital readmission. We did assess antimicrobial utilization in DOT/1000 patient days, but did not find a difference between groups, despite finding improvement in percentage of antimicrobials prescribed for the appropriate duration. This measure could have been confounded by the fact that many patients were discharged while still receiving antimicrobial therapy. Appropriate duration was determined by placement of antimicrobial stop dates. However, we were unable to control for or assess whether appropriate stop dates were added to discharge prescriptions.

There was a significant difference between the number of males and females in the pre- and post-intervention group as well as the number of patients with renal impairment and patients prescribed multiple antibiotics. Although logistic regression indicated that these differences had minimal effect on outcomes, effect of covariance cannot be ruled out. The quasi-experimental pre-post-intervention design can lead to difficulty in controlling for important confounding variables due to lack of randomization [13]. In some cases, providers were reluctant to accept recommendations if the antimicrobial was initiated by a previous provider. Furthermore, due to the use of weekly intervention, some antimicrobial orders were not reviewed by the project pharmacist. As the pre and post-intervention periods encompassed different times of the year, seasonal differences in infections, admissions, and antimicrobial prescribing could not be ruled out. Finally, our results apply to a specific population at a single institution, and may not be generalizable to all populations or institutions. However, this project does constitute an example of a potential practice for improving patient care.

### 4.3. Future Projects

Our project involved a once weekly pharmacist intervention. Analysis of the impact of daily pharmacist intervention could provide more insight into the effect on antimicrobial prescribing. Antibiotic inappropriateness can lead to many negative clinical consequences such as antimicrobial resistance, hospital readmission, and adverse drug reactions. Further investigation into the clinical impact, including readmission rates, of a similar intervention in geriatric psychiatric patients would be beneficial.
